# Screening for urinary bladder cancer with the use of nuclear matrix protein (NMP) 22: is it feasible?

**DOI:** 10.1186/1617-9625-12-S1-A6

**Published:** 2014-06-06

**Authors:** Anastasios Stefanopoulos, Konstantinos P Economopoulos

**Affiliations:** 1 Medical school, National and Kapodistrian University of Athens, Athens, 115 27, Greece; 2 Society of Junior Doctors, Athens,15123, Greece; 3 Massachusetts General Hospital, Harvard Medical School, Boston, Massachusetts, 02114, USA

## Background

Tobacco use accounts for the majority of urinary bladder cancer (UBC) cases both in men and women. It is widely accepted that smokers have increased risk of UBC [1-4]. An early diagnosis of UBC is crucial because patients with superficial UBC have much better prognosis than those with invasive UBC [1-5]. During the past years a variety of urine based markers have been introduced for screening of UBC. Nuclear matrix protein 22 (NMP22) is a nuclear protein that is accountable for chromatid regulation and cell separation during replication. The detection of NMP22 has been used from many clinical studies to evaluate the possibility of UBC screening [2-8]. The purpose of this review is to access the current literature in order to determine the usefulness of NMP22 in screening of UBC.

## Materials and methods

A thorough search was conducted in MEDLINE using the terms urinary bladder cancer OR urinary bladder neoplasm, NMP22 and screening. The following inclusion criteria were adopted: i. studies that NMP22 was measured in high risk patients without UBC and ii. studies that NMP22 was compared between patients with known history of UBC and a control group without history of UBC. We excluded studies that NMP22 was used only for the surveillance of UBC.

## Results

185 English-language articles were retrieved and 38 were included in this study. Average total sensitivity of NMP22 was 73.44 ± 15.11% and average total specificity 72.82 ± 16.27%. Positive predictive value (PPV) was 37.12 ± 26.11% and negative predictive value (NPV) was 87.47 ± 10.60%. Two studies did not report total specificity or sensitivity rates. The majority of the reports concluded than NMP22 cannot be used for screening non-invasive UBC but benefits the screening for high grade UBC in symptomatic patients. However there is no study in the literature that indicates that NMP22 detection approaches level 1 of evidence in screening for UBC. Based on the fact that the prevalence of UBC in asymptomatic high risk patients without history of UBC is low (4.0%), the diagnostic value of mass screening programs in asymptomatic patients is questionable.

## Conclusions

Detection of NMP22 is a non-invasive test that can be easily applied and gives diagnostic answers very quickly especially for tobacco-induced high grade tumors. NMP22 detection cannot replace cystoscopy. It is essential, more studies to be conducted with careful selection of patients, in order to find out, if NMP22 or a combination with other markers are useful for diagnosing UBC [1-5].
